# Public Perception of the Role and Professional Identity of Advanced Practice Nurses in Lithuania

**DOI:** 10.3390/nursrep16080267

**Published:** 2026-07-31

**Authors:** Andželika Zavackienė, Alina Štukova, Gintarė Venckauskytė, Asta Mažionienė

**Affiliations:** 1 Republic Klaipėda Hospital, S. Nėries g. 3, LT-92231 Klaipėda, Lithuaniavenc.gintare@gmail.com (G.V.); 2 Faculty of Health Sciences, Klaipėda State University of Applied Sciences, Bijūnų g. 10, LT-91223 Klaipėda, Lithuania; a.mazioniene@kvk.lt

**Keywords:** public perception, advanced practice nursing, professional identity

## Abstract

**Introduction/Objective:** Advanced practice nurses (APNs) play an increasingly important role in healthcare systems by expanding access to high-quality, patient-centred care. However, public understanding of the APNs’ role remains limited, particularly regarding their autonomy, leadership, and scope of practice. In Lithuania, APNs represent a relatively new professional group, and to date, no quantitative study has examined public perceptions of their role, competencies, and professional identity. Understanding these perceptions is essential for supporting the successful integration of APNs into the Lithuanian healthcare system. The aim of this study was to examine public perceptions of the role and professional identity of advanced practice nurses (APNs) in Lithuania. **Methods:** A quantitative cross-sectional research design was employed. Data were collected between October 2025 and January 2026 through an anonymous online questionnaire survey. Participants were recruited using a non-probability convenience sampling strategy. A total of 518 respondents participated in the study (*n* = 518). Data were collected using the Nursing’s Current Brand Position Scale (NCBPS) and the Nursing’s Desired Brand Position Scale (NDBPS). Data were analysed using descriptive statistics, correlation analysis, and principal component analysis (PCA). **Results:** The findings revealed that the public evaluates the professional image of advanced practice nurses (APNs) positively. However, it is primarily associated with traditional nursing values, particularly the perception of nursing as “a caring profession” and patient-centeredness. A statistically significant difference was found between the current and desired APN professional image across all evaluated dimensions (overall mean scores: 6.61 ± 1.67 vs. 7.68 ± 1.67; *t* = −18.426; *p* < 0.001)*,* with the largest gap observed in perceptions of APNs as patient advocates, educational leaders, and research contributors. The desired image reflects public expectations for APNs to function as autonomous, decision-making professionals who actively represent patients’ interests. A significant positive association was identified between professional image evaluation and trust in APN competencies (*r* = 0.66, *p* < 0.001). **Conclusions:** Participants in the present study generally perceived the professional image of advanced practice nurses (APNs) positively, primarily associating it with caring and patient-centred care rather than leadership and strategic roles. The comparison between the current and desired professional images suggests that respondents expressed higher expectations for the APN role. Respondents reported moderately high trust in APNs, which was significantly associated with perceptions of their professional image.

## 1. Introduction

Nursing is a dynamic profession whose capacity to respond to the evolving needs of society and healthcare systems has driven its continuous development. Over the past five decades, one of the most significant milestones in this development has been the emergence of advanced practice nurses (APNs), which has fundamentally transformed healthcare delivery models by expanding nurses’ responsibilities, competencies, and professional autonomy [1]. The role of APNs extends beyond traditional nursing functions. These professionals demonstrate leadership, provide independent expert consultations, deliver patient and public education, and actively collaborate with other healthcare professionals. Advanced practice nurses are employed across a broad range of healthcare settings, including primary healthcare, emergency care, anaesthesia, and critical care [2,3,4].

Although APNs possess extensive competencies and practise across diverse healthcare settings, their legal status and scope of practice vary considerably between countries. Consequently, public understanding of the APN role also differs across healthcare systems [2,4]. Public perceptions of advanced practice nurses influence the extent to which these professionals can be successfully integrated into healthcare services. Trust in APNs’ professional competence, their ability to provide high-quality care, and their overall professional reputation directly affect patients’ willingness to seek care from nursing professionals [5]. Previous studies have demonstrated that public attention towards the nursing profession increased during the COVID-19 pandemic. However, this period also highlighted insufficient public understanding of the responsibilities and scope of practice of advanced practice nurses [6,7]. The professional image of APNs is shaped not only through direct patient interactions but also through media representation, educational initiatives, and communication by healthcare institutions. In some countries, APNs are recognised as autonomous healthcare providers, whereas in others, they continue to be perceived primarily as assistants to physicians, influencing both their professional image and occupational prestige [8,9,10].

Advanced practice nursing has been formally established in Lithuania and is supported by a national strategic and legal framework. The National Nursing Policy Guidelines for 2016–2025 identify the development of advanced nursing practice, professional autonomy, and competency expansion as national healthcare priorities [11]. The Law on Nursing Practice and Midwifery Practice of the Republic of Lithuania (Law No. IX-413, 2001) and the Lithuanian Medical Norm MN 160:2026 “Advanced Practice Nurse” define the competencies, scope of practice, professional rights, and responsibilities of advanced practice nurses [12,13].

The 2023 preliminary audit report published by the National Audit Office of Lithuania identifies the strengthening of the advanced practice nursing workforce as one of the national priorities for healthcare system development. Nevertheless, limited educational capacity, insufficient professional recognition, and structural workplace challenges continue to restrict the establishment of APNs as autonomous and effective providers within primary healthcare. Furthermore, the role of advanced practice nurses remains insufficiently recognised and understood by the Lithuanian public [14].

As the advanced practice nurse role has only recently been introduced in Lithuania, it is essential to investigate how the public perceives the professional image and role of these specialists within the healthcare system. Although numerous international studies have examined the role and professional image of advanced practice nurses, there remains a lack of systematic evidence regarding public awareness and perceptions in the Lithuanian context [15,16]. This study is significant from both scientific and practical perspectives, as it seeks to address this knowledge gap by examining public perceptions of APNs’ competencies, professional role, and professional image. Public perception represents one of the key determinants of professional recognition, role clarity, and the successful integration of advanced practice nurses into healthcare systems. The findings of this study may contribute to evidence-informed policy and decision-making aimed at strengthening the position of advanced practice nurses and optimising both their current and potential contribution to healthcare system performance.

The aim of this study was to examine public perception of the role and professional identity of advanced practice nurses in Lithuania.

## 2. Materials and Methods

### 2.1. Research Procedure

A quantitative cross-sectional research design employing an anonymous questionnaire survey was applied in this study. Data were collected between October 2025 and January 2026 using an anonymous online questionnaire administered via the platform www.apklausa.lt.

Participants were recruited using a non-probability convenience sampling method. The questionnaire was disseminated through electronic communication channels. In addition, participants in healthcare institutions and patient organisations were invited to complete the questionnaire by scanning a QR code providing direct access to the online survey. Official requests for survey distribution were sent via e-mail to patient organisations and healthcare institutions. Following receipt of written permission, the electronic questionnaire was distributed through the Lithuanian Cancer Patient Coalition (Lietuvos onkologinių pacientų asociacija (POLA)), the Klaipėda County Multiple Sclerosis Society, and other patient organisations.

Individuals aged 18 years and older who were not employed within the healthcare sector were eligible to participate in the study. Eligibility was verified through automated screening questions regarding participants’ age and employment status before access to the questionnaire was granted. Individuals younger than 18 years of age and those employed within the healthcare sector were not eligible to participate. They were thanked for their interest and were not permitted to proceed with the questionnaire. Prior to data collection, the minimum required sample size was estimated to be 384 participants using a 95% confidence level, a 5% margin of error, and an assumed population proportion of 50%. The questionnaire was accessed 7780 times. A total of 596 questionnaires were submitted, of which 518 met the eligibility criteria, were complete, and were included in the final analysis.

### 2.2. Research Instruments

#### 2.2.1. Instrument Use, Adaptation, and Psychometric Evaluation

The Nursing’s Current Brand Position Scale (NCBPS) and the Nursing’s Desired Brand Position Scale (NDBPS) [10,17,18] were employed for data collection. Formal permission to use these instruments for research purposes was obtained from their developer, Dr Judi Allyn Godsey, together with the requirement to provide appropriate citations to the original sources [10,17,18]. Permission was granted to use the NCBPS with a public sample and to use the NDBPS with a public sample, with the notation that the psychometric properties of the NDBPS have not yet been validated within this population, as the instrument was originally designed to assess the professional image most desired by nurses for their own profession rather than perceptions held by the general public.

The developer further indicated that comparing the public’s current perceptions of advanced practice nurses, assessed using the NCBPS, with nurses’ desired professional image, assessed using the NDBPS, may help identify discrepancies between public perceptions and nurses’ preferred professional identity. Such comparisons may contribute to a better understanding of how closely public perceptions align with the professional image desired by nurses, particularly regarding leadership and caring attributes.

The wording of the instrument items was not modified during the study, in accordance with the developer’s recommendations. The questionnaires were translated from English into Lithuanian using the back-translation method to ensure linguistic accuracy and conceptual equivalence with the original versions of the instruments.

To evaluate the structure of the scales, principal component analysis (PCA) with Varimax orthogonal rotation was performed. Internal consistency of the scales and subscales was assessed by calculating Cronbach’s α coefficients. As the NCBPS and NDBPS instruments had not previously been applied to a public sample [10,17,18], factor analysis and reliability testing were performed to assess the structural suitability and psychometric properties of the instruments within the Lithuanian context.

#### 2.2.2. Structure of the Questionnaire

The first section comprised the Nursing’s Current Brand Position Scale (NCBPS), adapted to the context of advanced practice nursing, and included 10 items. This scale was designed to assess how the public currently perceives the role, prestige, and influence of advanced practice nurses (APNs) within the healthcare system. It enabled evaluation of the professional position of APNs in relation to other healthcare professionals and assessment of the current state of the professional image of APNs within society.

The second section comprised the Nursing’s Desired Brand Position Scale (NDBPS), also adapted to the context of advanced practice nursing, and included 10 items. The purpose of this scale was to determine respondents’ perceptions regarding the professional image and position that APNs should occupy within the healthcare system. The scale reflects the desired or ideal professional image of APNs, emphasising their expected role and influence in healthcare.

Items in the first and second sections of the questionnaire were assessed using a 10-point Likert-type scale, where 1 indicated “strongly disagree” and 10 indicated “strongly agree”. Higher scores reflected stronger agreement with the presented statements.

The third section of the questionnaire comprised six items assessing public trust in advanced practice nurses (APNs) and their professional competence. The items were developed by the authors based on a review of the scientific literature addressing patient trust in nurses [19], public perceptions of nursing competencies [20], the contribution of advanced practice nurses to healthcare quality, patient satisfaction, and clinical outcomes [21], and the professional prestige of nursing [22].

The literature review identified the main dimensions related to public trust in APNs, which formed the basis for developing the six questionnaire items. These dimensions included trust in APNs’ ability to manage healthcare needs, confidence in their professional advice, willingness to receive care from APNs as first-contact healthcare providers, preference for physician-led versus APN-led care, perceptions of APNs’ contribution to healthcare quality and patient satisfaction, and the professional prestige of nursing.

Accordingly, the first item assessed respondents’ trust in APNs’ ability to appropriately manage healthcare needs [19]. The second item evaluated confidence in healthcare advice provided by APNs compared with that provided by physicians [20]. The third item assessed willingness to have a health concern initially assessed by an APN [21]. The fourth (reverse-coded) item examined respondents’ preference for physician-led rather than APN-led care [20]. The fifth item evaluated perceptions of the contribution of APNs to healthcare quality and patient satisfaction [21]. The sixth item assessed perceptions of the professional prestige and societal respect associated with the nursing profession, including advanced practice nurses [22].

Together, these six items were designed to reflect the key dimensions of public trust in advanced practice nurses and their professional competence identified in the literature.

Responses were recorded using a 5-point Likert scale ranging from 1 “strongly disagree” to 5 “strongly agree”. A 5-point Likert scale was selected because it provides sufficient discrimination between response categories while remaining straightforward and easy for respondents to understand in population-based surveys.

The fourth section included respondents’ sociodemographic characteristics, including sex, age, place of residence, and educational attainment.

Prior to the main study, a pilot study was conducted to evaluate the clarity, comprehensibility, and content suitability of the questionnaire for the target population. Pilot participants were recruited according to the same inclusion criteria as those applied in the main study. Following the analysis of the first 17 completed questionnaires, the main point requiring clarification was the concept of the advanced practice nurse (APN). Therefore, the questionnaire was supplemented with a brief explanation of the professional role of the advanced practice nurse and a reference link to the valid Lithuanian Medical Norm MN 160:2026 “Advanced Practice Nurse” defining APN competencies [13]. Following these revisions, pilot testing continued until a total of 31 participants had completed the questionnaire. No further comments regarding the clarity or comprehensibility of the questionnaire were received. Data collected during the pilot study were not included in the final study sample or the statistical analysis.

### 2.3. Data Analysis

Data analysis was performed using IBM SPSS Statistics (version 27.0). The distribution of variables was assessed using the Shapiro–Wilk test and by evaluating skewness and kurtosis coefficients. Comparisons between two independent groups were conducted using the independent samples Student’s *t*-test (*t*), whereas comparisons among more than two groups were performed using the Kruskal–Wallis (*H*) test, depending on data distribution and group size. Comparisons between two related variables within the overall sample were analysed using the paired samples Student’s *t*-test. Associations between variables were evaluated using Pearson’s correlation coefficient (*r*) or Spearman’s rank correlation coefficient (*ρ*), depending on the distribution of the data. Analyses across sociodemographic groups were specified a priori as part of the study design to examine potential differences and associations in respondents’ perceptions of the current and desired professional image of advanced practice nurses. Results were considered statistically significant when *p* < 0.05. To examine the factorial structure of the scales, principal component analysis (PCA) with Varimax orthogonal rotation was applied. Varimax rotation was selected to achieve a simple and readily interpretable factor structure, consistent with the exploratory objective of the analysis. Internal consistency reliability of the scales and their factors was assessed using Cronbach’s alpha coefficients.

### 2.4. Ethical Aspects of the Research

This study was conducted in accordance with the principles of research ethics and internationally accepted ethical standards for research involving human participants, including the Declaration of Helsinki [23]. As the study employed an anonymous online survey of adult participants, did not involve any intervention, did not collect sensitive or personally identifiable information, and posed no more than minimal risk to participants. Therefore, formal approval from the national biomedical research ethics system was not required in accordance with Lithuanian regulations governing non-interventional anonymous surveys [24]. Nevertheless, the study protocol was reviewed by the Research Ethics Committee of the Faculty of Health Sciences, Klaipėda State University of Applied Sciences, Lithuania. The Committee confirmed that the study documentation complied with research ethics principles, that participants’ rights were protected, and raised no objections to the implementation of the study (Decision No. SSV5–18, 15 September 2025).

Prior to completing the questionnaire, participants were informed about the purpose of the study, the voluntary nature of participation, the assurance of anonymity and confidentiality, and their right to withdraw from the study at any time without providing a reason. Informed consent was considered to have been obtained when participants voluntarily proceeded to complete the online questionnaire. The questionnaire was fully anonymous, no personally identifiable information was collected, and all data were analysed and reported only in aggregated form for scientific purposes. Only adults (aged 18 years or older) were eligible to participate; therefore, no vulnerable populations were included in the study. To minimise any potential risks to participants, complete anonymity, confidential data handling, and the right to discontinue participation at any stage of the study were ensured.

## 3. Results

### 3.1. Characteristics of the Study Sample

A total of 518 respondents participated in the study (*n* = 518). Respondents’ age ranged from 19 to 79 years, with a mean age of 53.8 ± 15.3 years. The largest proportion of respondents belonged to the 60–69 years age group (29.0%). Most participants were female (76.6%), nearly half resided in major cities (49.6%), and the largest proportion had obtained a university-level higher education (EQF 6–8) degree (52.5%) (Table 1).

### 3.2. Current Professional Image of Advanced Practice Nurses (NCBPS)

Prior to conducting the factor analysis, the suitability of the data was assessed. The Kaiser–Meyer–Olkin (KMO) measure of sampling adequacy was excellent (KMO = 0.928), and Bartlett’s test of sphericity was statistically significant (χ^2^ = 4288.308, *p* < 0.001). The significant result of Bartlett’s test allowed the null hypothesis that the correlation matrix was an identity matrix to be rejected, indicating that sufficient intercorrelations existed among the variables to justify the application of factor analysis.

The NCBPS demonstrated excellent internal consistency, with a Cronbach’s alpha coefficient of 0.942, indicating a high level of internal reliability among the items. Principal component analysis identified a single-factor solution, which explained 66.02% of the total variance. All items exhibited communalities greater than 0.50, confirming that each item contributed adequately to the underlying factor structure (Table 2).

The findings suggest that the public perceives the current professional image of advanced practice nurses (APNs) as a unified construct. The overall mean score for the NCBPS was 6.61 ± 1.67 out of a maximum of 10, indicating a moderately favourable perception of the APN profession. Respondents rated APNs most highly as a caring profession (8.05 ± 2.00), followed by their focus on health and well-being (7.19 ± 2.01) and their patient-centred approach (7.09 ± 1.97). In contrast, the lowest ratings were assigned to APNs’ leadership in education, research, and clinical practice (5.51 ± 2.07) and their leadership in healthcare reform (5.81 ± 2.08).

### 3.3. Desired Professional Image of Advanced Practice Nurses (NDBPS)

The suitability of the data for factor analysis was confirmed by an excellent Kaiser–Meyer–Olkin (KMO) measure of sampling adequacy (KMO = 0.935) and a statistically significant Bartlett’s test of sphericity (χ^2^ = 5659.651, *p* < 0.001). These results indicate that the correlation matrix differed significantly from an identity matrix and that sufficient correlations existed among the variables to identify the underlying factor structure. The NDBPS demonstrated excellent internal consistency, with a Cronbach’s alpha coefficient of 0.955, confirming a high degree of homogeneity among the items. Principal component analysis (PCA) with Varimax rotation identified a two-factor solution, which together explained 82.45% of the total variance. All items exhibited communalities greater than 0.50, indicating that each item contributed adequately to the underlying factor structure.

The first factor, accounting for 71.68% of the total variance, comprised items related to caring, a patient-centred approach, holistic care, and patient advocacy. This factor was therefore interpreted as “Patient-Centred Advocates” and demonstrated excellent internal consistency (Cronbach’s α = 0.956). The second factor explained an additional 10.77% of the total variance and included items reflecting leadership, decision-making, professional autonomy, and healthcare reform. Accordingly, this factor was interpreted as “Influential Leaders” (Cronbach’s α = 0.922) (Table 3).

Mean scores were calculated for both the overall desired professional image of advanced practice nurses (NDBPS) (7.68 ± 1.67) and for each extracted factor separately. The analysis revealed that respondents rated the desired professional image of APNs significantly higher as patient-centred advocates (8.02 ± 1.72) than as influential leaders (6.97 ± 1.88).

The mean scores of the individual NDBPS items ranged from 6.86 ± 2.03 to 8.48 ± 1.82. Among the desired professional image attributes, respondents assigned the highest ratings to APNs as a caring profession (8.48 ± 1.82), APNs as influential patient advocates (8.27 ± 1.94), and APNs adopting a patient-centred approach (8.24 ± 1.84).

### 3.4. Comparison of the Current Professional Image (NCBPS) and Desired Professional Image (NDBPS) of Advanced Practice Nurses

The study findings indicated that the ratings obtained with the current professional image of advanced practice nurses (NCBPS) were significantly lower than those obtained with the desired professional image of advanced practice nurses (NDBPS) across all evaluated dimensions. (Table 4). Statistically significant differences were observed between the current and desired professional images of advanced practice nurses across all analysed indicators (*p* < 0.001).

The largest differences between the current (NCBPS) and desired (NDBPS) professional images of advanced practice nurses were observed for APNs as influential patient advocates (NCBPS: 6.66 ± 2.21; NDBPS: 8.27 ± 1.94; *t* = −16.821; *p* < 0.001), leaders in education, research and clinical practice (NCBPS: 5.51 ± 2.07; NDBPS: 6.87 ± 2.02; *t* = −15.951; *p* < 0.001), and leaders in healthcare reform (NCBPS: 5.81 ± 2.08; NDBPS: 7.12 ± 2.12; *t* = −14.800; *p* < 0.001).

The ratings obtained with the NDBPS were significantly higher than those obtained with the NCBPS. The overall difference between the two scales was also statistically significant (NCBPS: 6.61 ± 1.67; NDBPS: 7.68 ± 1.67; *t* = −18.426; *p* < 0.001).

The overall ratings of the current and desired professional image of advanced practice nurses (APNs) did not differ significantly between female and male respondents. However, significant gender differences were observed for several individual attributes. Compared with men, women rated the current image of APNs significantly lower as empowered decision-makers (women: 6.06 ± 2.03; men: 6.52 ± 1.92; *t* = −2.210; *p* = 0.028) and as leaders in education, research and clinical practice (women: 5.40 ± 2.04; men: 5.88 ± 2.13; *t* =−2.249; *p* = 0.025). Conversely, women expressed significantly stronger expectations that APNs should provide holistic healthcare to patients and the public (women: 7.96 ± 2.00; men: 7.54 ± 2.07; *t* = 2.045; *p* = 0.041) and should act as influential patient advocates (women: 8.37 ± 1.90; men: 7.93 ± 2.03; *t* =2.179; *p* = 0.030).

The overall ratings of the current and desired professional image of APNs also did not differ significantly according to respondents’ place of residence. Nevertheless, significant differences were identified for several individual attributes. Respondents living in major cities rated APNs significantly lower as leaders in education, research and clinical practice (major cities: 5.19 ± 2.05; suburban area of a major cities: 5.90 ± 1.92; small cities/towns: 5.82 ± 2.12; rural areas: 5.78 ± 1.90; H = 14.106; *p* = 0.003) and as leaders in healthcare reform (major cities: 5.50 ± 2.10; suburban areas of a major cities: 6.27 ± 2.02; small cities/towns: 6.18 ± 2.05; rural areas: 5.93 ± 1.95; H = 12.787; *p* = 0.005) than respondents residing in smaller communities.

A significant difference in the desired image of APNs as empowered decision-makers was also observed according to place of residence. This attribute was least strongly endorsed by respondents living in major cities and most strongly endorsed by those living in metropolitan suburbs (major cities: 6.69 ± 2.08; suburban areas of major cities: 7.97 ± 1.73; small cities/towns: 6.92 ± 1.91; rural areas: 6.92 ± 2.07; H = 9.701; *p* = 0.021).

Analysis of the individual dimensions of the current professional image of advanced practice nurses (APNs) revealed significant age-related differences. Respondents aged 40–49 years and 19–29 years rated APNs most favourably with respect to evidence-based practice (19–29 years: 7.20 ± 2.12; 30–39 years: 6.63 ± 1.98; 40–49 years: 7.21 ± 1.93; 50–59 years: 6.18 ± 2.03; 60–69 years: 6.48 ± 1.83; ≥70 years: 6.58 ± 1.99; H = 13.883; *p* = 0.016), a focus on health and well-being (7.82 ± 2.03; 7.29 ± 2.19; 8.02 ± 1.89; 6.58 ± 2.01; 7.05 ± 1.77; 7.22 ± 2.05, respectively; H = 28.597; *p* < 0.001), a patient-centred approach (7.60 ± 2.04; 7.22 ± 2.06; 7.73 ± 1.72; 6.66 ± 1.96; 6.93 ± 1.85; 7.08 ± 2.11, respectively; H = 17.418; *p* = 0.004), and nursing as a caring profession (8.36 ± 2.12; 8.04 ± 2.13; 8.73 ± 1.71; 7.72 ± 2.07; 8.05 ± 1.78; 7.83 ± 2.22, respectively; H = 14.932; *p* = 0.011). In contrast, respondents aged 50–59 years consistently assigned the lowest ratings to these attributes.

Regarding APNs as empowered decision-makers (6.13 ± 1.56; 5.49 ± 2.35; 6.58 ± 1.97; 6.08 ± 2.00; 6.14 ± 1.93; 6.68 ± 1.96, respectively; H = 15.045; *p* = 0.010) and as providers of holistic healthcare to patients and the public (6.80 ± 2.12; 6.44 ± 2.29; 7.45 ± 2.06; 6.61 ± 1.99; 6.93 ± 1.84; 7.14 ± 2.08, respectively; H = 14.327; *p* = 0.014), the highest ratings were reported by respondents aged 40–49 years and 70 years or older, whereas respondents aged 30–39 years assigned the lowest ratings.

The current professional image of advanced practice nurses (APNs) (overall score) was rated most favourably by respondents with vocational (EQF 4–5) and college level education (EQF 5) and least favourably by those with primary (EQF 2)/secondary education (EQF 4) and a master’s (EQF 7) or doctoral degree (EQF 8) (6.28 ± 2.20, 6.90 ± 1.53, 6.86 ± 1.56, 6.63 ± 1.52, and 6.30 ± 1.76 points, respectively; H = 10.266; *p* = 0.036). No significant differences in the ratings of the desired professional image of advanced practice nurses were observed across age groups. Spearman’s rank correlation analysis also revealed no statistically significant association between respondents’ educational level and the overall professional image score of advanced practice nurses (*ρ* = −0.07, *p* = 0.096).

Respondents with vocational and college education rated several aspects of the current professional image of APNs more favourably than respondents in the other educational groups, particularly those with a master’s (EQF 7) or doctoral degree (EQF 8) and those with primary (EQF 2)/secondary education (EQF 4). These aspects included APNs as leaders in healthcare reform (5.68 ± 2.55, 6.21 ± 2.09, 6.04 ± 2.08, 5.84 ± 1.86, and 5.40 ± 2.06 points, respectively; H = 11.265; *p* = 0.024), providers of holistic healthcare for patients and society (6.44 ± 2.44, 7.12 ± 1.85, 7.18 ± 2.09, 6.92 ± 1.80, and 6.57 ± 2.14 points, respectively; H = 9.865; *p* = 0.043), and influential patient advocates (6.39 ± 2.50, 7.12 ± 2.01, 7.13 ± 2.11, 6.60 ± 2.06, and 6.15 ± 2.32 points, respectively; H = 17.717; *p* = 0.001).

### 3.5. Public Trust in Advanced Practice Nurses (APNs) and Their Professional Competence

When analysing public trust in advanced practice nurses (APNs) and their professional competence, the scale demonstrated good internal consistency, with a Cronbach’s alpha coefficient of 0.792, indicating satisfactory internal reliability among the items (Table 5).

The study findings showed that the overall level of public trust in advanced practice nurses (APNs) and their professional competence was moderately high, with a mean score of 3.62 ± 0.73 out of a maximum of 5. Respondents assigned the highest ratings to the statement that integrating APNs into healthcare teams can improve the quality of healthcare services and increase patient satisfaction (4.13 ± 0.90), followed by the statement that the nursing profession, including advanced practice nurses, is prestigious and respected within society (4.05 ± 1.02).

The study findings demonstrated that respondents residing in metropolitan suburbs reported the highest level of trust in advanced practice nurses (APNs), whereas those living in major cities reported the lowest level of trust (major cities: 3.55 ± 0.74; suburban area of a major cities: 3.99 ± 0.54; small cities/towns: 3.65 ± 0.75; rural areas: 3.67 ± 0.66; H = 10.114; *p* = 0.017). Respondents living in metropolitan suburbs were significantly more likely than those residing in other areas, particularly major cities, to agree that an advanced practice nurse should be the first healthcare professional to assess their health concerns (major cities: 3.24 ± 1.21; suburban area of a major cities: 3.97 ± 0.85; small cities/towns: 3.48 ± 1.19; rural areas: 3.49 ± 1.05; H = 12.186; *p* = 0.007).

No statistically significant differences in the overall trust score for advanced practice nurses (APNs) and their professional competence were observed across respondents’ age groups. A significant age-related difference was identified only for the reverse-coded statement, “I would always prefer to consult a physician rather than an advanced practice nurse to address my health concerns” (19–29 years: 2.96 ± 1.07; 30–39 years: 2.73 ± 1.23; 40–49 years: 2.71 ± 1.15; 50–59 years: 2.41 ± 1.06; 60–69 years: 2.32 ± 1.14; ≥70 years: 2.47 ± 1.22; H = 16.920; *p* = 0.005).

The overall trust score in APNs and their professional competence did not differ significantly according to respondents’ educational level. Statistically significant differences were identified only for two items: trust in APNs’ ability to provide appropriate healthcare (H = 9.978; *p* = 0.041) and perceptions of the prestige of the nursing profession (H = 13.518; *p* = 0.009). In both cases, the highest ratings were reported by respondents with vocational (EQF 4–5) or college education (EQF 5), whereas the lowest ratings were given by respondents holding master’s (EQF 7) or doctoral (EQF 8) degrees.

The study findings demonstrated that more favourable evaluations of both the current (*r* = 0.66, *p* < 0.001) and desired (*r*= 0.60, *p* < 0.001) professional image of advanced practice nurses (APNs) were significantly associated with higher levels of public trust in APNs and their professional competence. Furthermore, nearly all dimensions of both professional image scales were strongly and positively correlated (*p* < 0.001) with almost all dimensions of the trust scale, with the exception of the reverse-coded item, “I would always prefer to consult a physician rather than an advanced practice nurse to address my health concerns”.

## 4. Discussion

The findings of this study revealed a statistically significant discrepancy between the public’s perception of the current and desired professional image of advanced practice nurses (APNs). This pattern is consistent with previous studies indicating that the public image of the nursing profession often fails to reflect the full scope of nurses’ competencies and the complexity of their professional role [1,5,6]. These findings suggest the existence of a structural gap between the evolution of the profession and public perceptions of advanced practice nursing.

The lowest ratings of the current professional image of APNs were observed in domains related to leadership in education and research, as well as participation in decision-making processes. These findings are consistent with those reported by Godsey et al., who found that the public more commonly associates the nursing profession with caring and direct patient care than with leadership, academic responsibilities, or strategic roles within healthcare systems [10]. In the present study, the validated NCBPS and NDBPS instruments, both originally developed by Godsey et al. [10], were used to examine respondents’ perceptions of the current and desired professional image of advanced practice nurses. Together, they provided complementary information on these two dimensions of professional image.

Respondents perceived advanced practice nurses (APNs) as “patient advocates” and nursing as “a caring profession”. This finding is consistent with those reported by Duan et al. and Ziegler et al., who described the nursing profession as being primarily characterised by empathy, patient-centred care, and therapeutic relationships [7,8]. However, the significant discrepancy identified between the current and desired professional images suggests that respondents in the present study expected a broader role for advanced practice nurses. Although the traditional values of nursing remain highly important, public expectations extend to greater professional autonomy, increased involvement in clinical decision-making, and stronger leadership within the healthcare system. This expectation is consistent with international models of advanced practice nursing, which emphasise professional autonomy, clinical leadership, and evidence-based practice [2,3,4].

Nevertheless, uncertainty regarding the definition and scope of advanced practice nursing roles persists internationally, as role classifications and competency frameworks continue to vary across countries [15]. These findings suggest that public expectations appear to be aligned with international standards of advanced practice nursing. However, public understanding and recognition of the APN role have not yet been fully established.

An important finding of this study was the strong positive correlation between perceptions of the professional image of advanced practice nurses (APNs) and public trust in their professional competence. This finding is consistent with the proposition advanced by Godsey et al. and Moghbel et al. that professional image and public trust are closely interconnected [5,10]. The more favourably the professional role of APNs was perceived, the greater the level of public trust in their competencies. These findings suggest that a more favourable professional image may be associated with greater recognition and acceptance of advanced practice nurses as autonomous healthcare professionals, which may, in turn, support their integration within the healthcare system.

It is noteworthy that the only trust-related item that was not significantly associated with most dimensions of the professional image scales was the preference to consult a physician rather than an Advanced Practice Nurse (APN) as the first point of contact for health concerns. This finding may be interpreted in light of the concepts of professional hierarchy and medical dominance, which have historically positioned physicians as the principal decision-makers within healthcare systems [8,9].

Public preference for consulting a physician may not necessarily reflect differences in clinical outcomes or objective assessments of professional competence. Instead, it may be influenced by historically established professional power structures, limited recognition of the APN role, and persistent cultural stereotypes that continue to hinder the acceptance of advanced practice nurses as autonomous healthcare professionals.

However, systematic reviews and international evidence consistently demonstrate that the clinical outcomes of care provided by advanced practice nurses are comparable to, and in some cases better than, those achieved through physician-led care. Moreover, the integration of advanced practice nurses into healthcare services has been associated with improved management of chronic diseases, lower rates of initial and recurrent hospitalisations, and higher levels of patient satisfaction with healthcare services [25,26,27].

International evidence indicates that a clear legal framework and a well-defined system of professional accountability are fundamental prerequisites for strengthening APNs’ autonomy and clarifying the scope of advanced nursing practice [21]. This is consistent with the findings of the present study, which demonstrated a significant positive association between perceptions of APNs’ professional image and public trust in their competencies. Taken together, the available evidence and the findings of the present study suggest that institutional and regulatory conditions may indirectly strengthen public trust by improving the clarity and visibility of the APN role. In Lithuania, the development of advanced practice nursing has been recognised as a national healthcare priority [14].

The National Nursing Policy Guidelines for 2016–2025 identify the development of advanced nursing practice, professional autonomy, and competency expansion as strategic priorities [11]. The Law on Nursing Practice and Midwifery Practice of the Republic of Lithuania and the Lithuanian Medical Norm MN 160:2026 “Advanced Practice Nurse” define the legal framework governing APN practice, including the scope of practice, professional competencies, rights, and responsibilities of APNs [12,13].

Although advanced practice nurses have been integrated into the Lithuanian healthcare system, the implementation of their role in routine clinical practice continues to face challenges [28]. Despite the existing legal framework, limited professional recognition and structural challenges within healthcare organisations continue to restrict the full utilisation of APNs’ competencies. Taken together with existing evidence, these findings also highlight the need for continued development of the national nursing policy to strengthen APN career pathways, competency development, and professional support mechanisms, thereby facilitating the long-term integration and recognition of the APN role in Lithuania [11,14,28].

These barriers may contribute to the discrepancy between the current and desired professional image of APNs observed in the present study. Consequently, targeted communication emphasising APNs’ competencies, professional autonomy, and contribution to healthcare system performance may strengthen both their professional status and public trust.

One limitation of this study is related to participant recruitment. Although convenience sampling was used and the survey was distributed online through multiple channels to achieve an adequate sample size, recruitment also involved patient organizations. Consequently, some participants may have had more frequent contact with the healthcare system than the general population, which may have introduced selection bias. Therefore, the findings should be interpreted with appropriate caution, as they may not be fully representative of the broader Lithuanian population.

A further limitation of this study relates to the Desired Brand Image Scale (NDBPS). Although the psychometric properties of the instrument were evaluated in the present study, the NDBPS had not previously been validated for use in a public population, as it was originally developed to assess the professional image desired by nurses for their own profession. In addition, confirmatory factor analysis was not performed. Therefore, the factorial structure identified in the present study requires confirmation in future studies using independent public samples. Accordingly, findings related to the desired professional image of advanced practice nurses should be interpreted with appropriate caution.

Future studies using representative samples are needed to improve the generalisability of the findings. In addition, further validation of the NDBPS in independent public samples, including confirmatory factor analysis, is warranted. Finally, additional studies are needed to confirm the findings of the present study in different populations and settings.

## 5. Conclusions

The findings of this study indicate that respondents generally perceived the professional image of advanced practice nurses (APNs) positively, primarily associating it with traditional nursing values such as caring and patient-centredness. However, respondents did not fully recognise key APN competencies, including autonomous decision-making, leadership, and strategic contributions to the healthcare system. At the same time, respondents expressed clear expectations that APNs should provide holistic care, actively advocate for patients, and contribute to improving the quality of healthcare services.

The comparison between the two scales suggests that respondents expressed higher expectations than their ratings of the current professional image of the APN role in Lithuania. Respondents reported moderately high trust in APNs, which was closely associated with perceptions of their professional image. Taken together with existing evidence, the findings of this study suggest that strengthening public awareness of APN competencies may contribute to their professional recognition and institutional integration of APNs within the Lithuanian healthcare system.

## Figures and Tables

**Table 1:** Sociodemographic characteristics of the study participants (*n* = 518).

Variables	*n* = 518
Age, years, mean ± SD	53.80 ± 15.29
Age, *n* (%)	
19–29 years	45 (8.7%)
30–39 years	73 (14.1%)
40–49 years	62 (12.0%)
50–59 years	111 (21.4%)
60–69 years	150 (29.0%)
≥70 years	77 (14.9%)
Sex, *n* (%)	
Female	397 (76.6%)
Male	121 (23.4%)
Place of residence, *n* (%)	
Major city	257 (49.6%)
Suburban area of a major city	30 (5.8%)
Small city/town	157 (30.3%)
Rural area	74 (14.3%)
Educational attainment, *n* (%)	
Primary education (EQF 2) *	4 (0.8%)
Secondary education (EQF 4) *	37 (7.1%)
Vocational education (EQF 4–5) *	85 (16.4%)
College-level education (EQF 5) *	120 (23.2%)
Bachelor’s degree (EQF 6) *	129 (24.9%)
Master’s degree (EQF 7) *	138 (26.6%)
Doctoral degree (EQF 8) *	5 (1.0%)

Note: Data in the table are presented as mean scores ± standard deviations (SD). *—Educational attainment categories correspond to the European Qualifications Framework (EQF).

**Table 2:** Factor Structure and Psychometric Properties of the Current Professional Image of Advanced Practice Nurses Scale (NCBPS).

No.	Statement	Factor Loading	Mean ± SD
1	Empowered decision makers	0.771	6.17 ± 2.01
2	Leaders in education, research and practice	0.781	5.51 ± 2.07
3	Base practice on evidence/science	0.816	6.60 ± 1.98
4	Leaders in healthcare reform	0.827	5.81 ± 2.08
5	Autonomous healthcare providers	0.838	6.11 ± 2.24
6	Focus on health/wellness	0.859	7.19 ± 2.01
7	Patient-centred approach	0.869	7.09 ± 1.97
8	Caring profession	0.709	8.05 ± 2.00
9	Holistic healthcare for patients and the public	0.805	6.88 ± 2.04
10	Influential patient advocates	0.838	6.66 ± 2.21
	Overall NCBPS score ± SD	-	6.61 ± 1.67

Note: Data are presented as mean ± SD. Mean scores are presented on a 10-point scale. Principal component analysis was used for factor extraction. As only one factor was extracted, no rotation method was applied.

**Table 3:** Factor Structure and Psychometric Properties of the Desired Professional Image of Advanced Practice Nurses Scale (NDBPS).

Factor	Explained Variance (%)	Cronbach’s α	Mean ± SD	Attributes
Factor 1. Patient-Centred Advocates	71.68	0.956	8.02 ± 1.72	Base practice on evidence/science (0.702) Focus on health/wellness (0.849) Patient-centred approach (0.895) Caring profession (0.910) Holistic healthcare for patients and the public (0.767) Influential patient advocates (0.835)
Factor 2. Influential Leaders	10.77	0.922	6.97 ± 1.88	Empowered decision makers (0.833) Leaders in education, research and practice (0.865) Leaders in healthcare reform (0.807) Autonomous healthcare providers (0.807)
Overall NDBPS score ± SD	-	-	7.68 ± 1.67	-

Note: Data are presented as mean ± SD. Mean scores are presented on a 10-point scale. Factors were extracted using principal component analysis with Varimax rotation. Factor loadings are presented in parentheses.

**Table 4:** Comparison Chart: Current (NCBPS) and Desired (NDBPS) Professional Image of Advanced Practice Nurses.

No.	Statement	NCPBS	NDPBS	t	*p*
1	Empowered decision makers	6.17 ± 2.01	6.86 ± 2.03	−8647	<0.001
2	Leaders in education/research/practice	5.51 ± 2.07	6.87 ± 2.02	−15,951	<0.001
3	Base practice on evidence/science	6.60 ± 1.98	7.86 ± 1.96	−15,229	<0.001
4	Leaders in healthcare reform	5.81 ± 2.08	7.12 ± 2.12	−14,800	<0.001
5	Autonomous healthcare providers	6.11 ± 2.24	7.03 ± 2.17	−10,778	<0.001
6	Focus on health/wellness	7.19 ± 2.01	8.17 ± 1.87	−11,722	<0.001
7	Patient-centred approach	7.09 ± 1.97	8.24 ± 1.84	−14,394	<0.001
8	Caring profession	8.05 ± 2.00	8.48 ± 1.82	−6187	<0.001
9	Holistic healthcare to patients/public	6.88 ± 2.04	7.86 ± 2.02	−11,970	<0.001
10	Influential patient advocates	6.66 ± 2.21	8.27 ± 1.94	−16,821	<0.001
	Overall mean score ± SD	6.61 ± 1.67	7.68 ± 1.67	−18,426	<0.001

Note: Data are presented as mean ± SD. Mean scores are presented on a 10-point scale. *t*, paired Student’s *t*-test statistic; *p*, significance level. Significant differences (*p* < 0.05) are presented in bold.

**Table 5:** Mean Scores for Public Trust in Advanced Practice Nurses (APNs) and Their Professional Competence.

No.	Statement		Mean ± SD
1	I trust that an advanced practice nurse (APN) can appropriately manage my healthcare needs [19]		3.85 ± 0.95
2	If an advanced practice nurse (APN) provided me with healthcare advice, I would trust it as much as advice from a physician [20]		3.81 ± 1.02
3	If given the opportunity, I would agree to have my health concern initially assessed by an advanced practice nurse (APN) [21]		3.39 ± 1.18
4 *	I would always prefer to consult only a physician rather than an advanced practice nurse (APN) to address my health concerns [20]		2.52 ± 1.16
5	Including advanced practice nurses (APNs) in the healthcare team can improve the quality of services provided and increase patient satisfaction with healthcare services [21]		4.13 ± 0.90
6	I believe that the nursing profession (including advanced practice nurses) is prestigious and respected in society [22]		4.05 ± 1.02
		Overall mean score ± SD	3.62 ± 0.73

Note: Data are presented as mean ± SD. Mean scores are presented on a 5-point scale. *—responses to items marked with an asterisk were reverse-coded.

## Data Availability

The data presented in this study are available from the corresponding author upon reasonable request. The data are not publicly available because of privacy and ethical considerations.

## Public Involvement Statement

Members of the public were not involved in the design, conduct, reporting, or dissemination plans of this research.

## Guidelines and Standards Statement

This manuscript was prepared in accordance with the Strengthening the Reporting of Observational Studies in Epidemiology (STROBE) Statement for cross-sectional studies [29].

## Use of Artificial Intelligence

During the preparation of this manuscript, ChatGPT (GPT-5.6 Sol Pro; OpenAI, San Francisco, CA, USA) was used to assist with translation from Lithuanian into English, language editing, improve-ment of academic writing style and clarity, reference formatting in accordance with the target jour-nal’s requirements, and preparation of the graphical abstract. Grammarly for Windows and Mac (version 1.2.258.1885; Grammarly, San Francisco, CA, USA) was used for grammar and spelling cor-rection. All AI-assisted outputs, including the graphical abstract, were carefully reviewed, verified, and edited by the authors, who take full responsibility for the final content of the manuscript. No AI tools were used for study design, data collection, statistical analysis, interpretation of the findings, or formulation of the scientific conclusions.
